# Halide Double-Perovskite Semiconductors beyond Photovoltaics

**DOI:** 10.1021/acsenergylett.2c00811

**Published:** 2022-05-31

**Authors:** Loreta A. Muscarella, Eline M. Hutter

**Affiliations:** Department of Chemistry, Utrecht University, Princetonlaan 8, 3584 CB Utrecht, The Netherlands

## Abstract

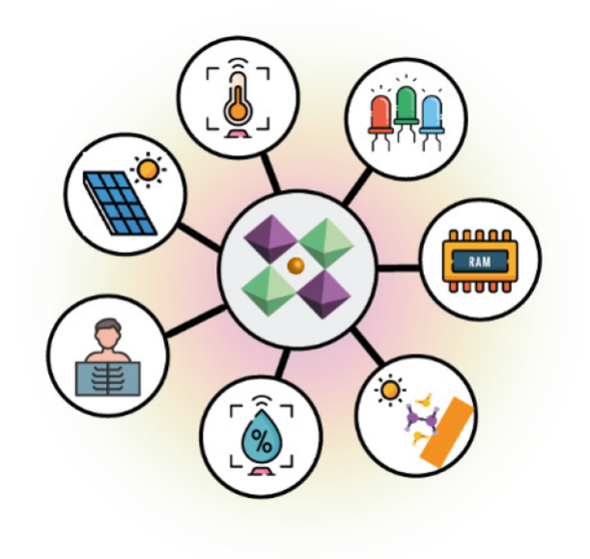

Halide double perovskites, A_2_M^I^M^III^X_6_, offer a vast chemical space for obtaining unexplored materials with exciting properties for a wide range of applications. The photovoltaic performance of halide double perovskites has been limited due to the large and/or indirect bandgap of the presently known materials. However, their applications extend beyond outdoor photovoltaics, as halide double perovskites exhibit properties suitable for memory devices, indoor photovoltaics, X-ray detectors, light-emitting diodes, temperature and humidity sensors, photocatalysts, and many more. This Perspective highlights challenges associated with the synthesis and characterization of halide double perovskites and offers an outlook on the potential use of some of the properties exhibited by this so far underexplored class of materials.

Halide double perovskites, officially called *elpasolites*, constitute a class of quaternary materials sharing the general formula A_2_M^I^M^III^X_6_ and have been known for more than a century. This class of materials has recently gained new interest when researchers from the halide perovskite community proposed the semiconductor Cs_2_AgBiBr_6_ as an alternative material with reduced toxicity compared to widely investigated lead-based perovskites such as MAPbI_3_ (MA = methylammonium) or CsPbBr_3_.^1−3^ Another advantage of halide double perovskites over common lead halide perovskites is their higher stability under ambient conditions and high-intensity illumination.^4^ The elpasolite crystal structure is similar to the perovskite lattice (Figure 1a) but contains octahedra with a monovalent M^I^ (1+) and a trivalent M^III^ (3+) cation instead of Pb^2+^, which are ordered in an alternating fashion. A related crystal structure is the so-called vacancy-ordered perovskite, alternating a quadrivalent (4+) cation and a vacancy at the M-site. Given that the halide double perovskites contain two metals instead of one, this class of materials offers a huge variety of compositions,^5−7^ where in principle any combination should be possible, provided that the tolerance factor and the octahedral factor are satisfied.^8^ However, satisfying the geometrical constraints of the tolerance and the octahedral factor alone does not ensure thermodynamic stability against decomposition. High-throughput first-principles calculations^9^ of the convex hull energy (i.e., Gibbs free energy of the compounds at zero temperature) of halide double perovskites with respect to decomposition products reveal that the predicted stability of a composition against its decomposition in byproducts is heavily affected by the size of A, X, and M^I^ elements, with minor effects from the size of M^III^. Higher stability can be achieved using larger A^+^ cations (e.g., Cs^+^ is preferred over Li^+^) and smaller halides (e.g., F^–^ is preferred over I^–^), as predicted from calculations on the lead-based counterpart. The stability trend as a function of the M^I^ size varies with the group of the periodic table (e.g., Ag^+^ is preferred over Cu^+^). These calculations do not account for geometrical factors and do not include the effects of entropy and pressure. However, estimating the formation energy (consisting of the atomization enthalpy, the ionization enthalpy, and the lattice enthalpy) of the desired composition is a powerful approach to predict its thermodynamic stability against phase decomposition into other compounds.^10^ For the lead-based perovskites, the stability of a composition increases when moving from iodide to chloride due to an increase in the ionization energy of the [PbX_6_]^−^ inorganic cage,^11^ similarly to the trend of the convex hull energy. However, to assess the thermodynamic stability against decomposition of such halide double perovskites, further computational investigations should be performed to evaluate the three terms of the formation energy, especially to account for the presence of two types of metals. The valence band maximum (VBM) of halide double perovskites is dominated by bonding orbitals of M^III^ (*n*s), M^I^ (*n*d), and X (*n*p), whereas the conduction band minimum (CBM) is formed by antibonding orbitals M^III^ (*n*p) and X (*n*p).^12^ This is similar to most covalent semiconductors, such as silicon or germanium, and group III–V or II–VI semiconductors, like GaAs and CdSe, respectively. Notably, in the lead halide perovskites, both the VBM and CBM are composed of antibonding orbitals, so that many crystallographic defects do not initiate an electronic (trap) state in the bandgap. It is therefore likely that the halide double perovskites are not as defect-tolerant as their lead-based analogues, meaning that more effort is needed to make these materials defect-poor.

**Figure 1:**
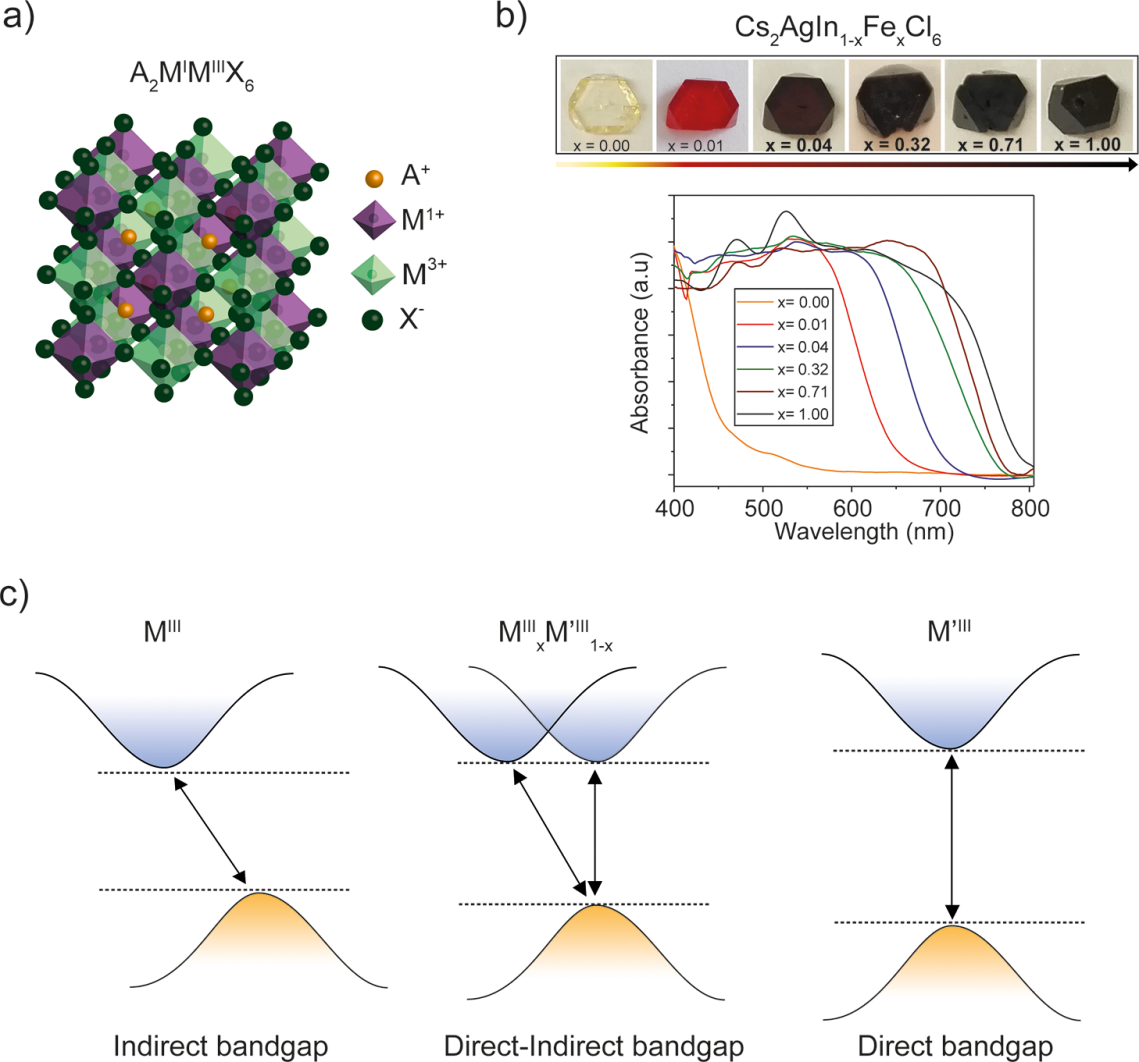
(a) Crystal structure of halide double perovskites described by the formula A_2_M^I^M^III^X_6_ , consisting of alternating corner-sharing [M^I^X_6_] and [M^III^X_6_] octahedra shown in purple and green, respectively. Orange and dark green spheres represent the monovalent cation A^+^ (often Cs^+^) and the halide X^–^, respectively. (b) Photographs and normalized UV–Vis absorption spectra of Cs_2_AgIn_1-*x*_Fe_*x*_Cl_6_ (*x* = 0.00, 0.01, 0.04, 0.32, 0.71, and 1.00) crystals. Adapted from ref (17). Copyright 2021 Royal Society of Chemistry. (c) Schematic representation of the halide double-perovskite band structure, showing changes in the magnitude (energy) and nature (indirect and direct) of the bandgap upon mixing different trivalent metals, M^III^ and M′^III^.

Most of the experimentally reported halide double perovskites contain chloride or bromide at the halide site, which have larger bandgaps than their iodide analogues. Incorporating iodide is more challenging because the size mismatch with the relatively small trivalent cations limits the geometric stability.^8^ Lanthanides (e.g., Ce^3+^, La^3+^) could be at the M^III^ site used to meet the radius ratio criterion and favor the formation of iodide-based double perovskites.^13^ However, some of the bromide- and even chloride-based double perovskites show suitable bandgaps for photovoltaics (PV) applications,^14^ such as Cs_2_AgFeCl_6_ (experimental bandgap of ∼1.55 eV)^15^ and predicted compositions like Cs_2_AgGaBr_6_ (expected bandgap of ∼1.37 eV). Precise bandgap tunability can be obtained when making solid solutions of halide double perovskites, in which mixtures of trivalent metals result in materials lattice parameters and bandgaps intermediate between those of the “parent compounds”.^16,17^Figure 1b shows an example of a mixed indium–iron double-metal perovskites, showing a substantial red-shift of the absorption already at 1% iron.^17^ Interestingly, in solid solutions, not only the magnitude but also the nature of the bandgap can be tuned from direct to indirect and in between (Figure 1c).^18^ Therefore, mixing trivalent metals in halide double perovskites can serve as a strategy to design materials with direct–indirect bandgaps. Such rationally designed direct–indirect semiconductors could be useful to combine strong absorption, driven by direct absorption transitions, with slow recombination, characteristic for indirect bandgaps.^13,18,19^ Semiconductors with such extensive tunability are in sharp contrast with silicon, which always exhibits an indirect bandgap and does not allow for bandgap tunability, unless it is nanostructured. On the other hand, III–V semiconductors such as GaAs do offer tunability of the magnitude and nature of the bandgap when gallium or arsenic atoms are partially replaced by other elements from the same group. However, the narrow choice of elements in groups III–V limits the achievable combinations of magnitude and nature of the bandgap. Besides, many III–V semiconductors contain scarce elements, while halide double perovskites could, in principle, be made using more abundant elements. So far, the PV community has mainly focused on Cs_2_AgBiBr_6_, which, despite efforts, still shows poor performance in solar cells (i.e., PCE < 3%).^20^ This is in part due to the intrinsic limitation of weak sunlight absorption, because of the large (2.2 eV) and indirect bandgap of Cs_2_AgBiBr_6_. On the other hand, the performance of Cs_2_AgBiBr_6_ has been more encouraging in other applications, such as X-ray detectors,^21,22^ due to its strong X-ray absorption. Finally, this class of materials has potential in the field of photocatalysis,^23,19^ where large bandgaps may be necessary to drive certain photoredox reactions, or indoor photovoltaics, where the incident light matches better with the absorption spectrum.

In comparison with lead halide perovskites, there are several challenges associated with solution-processed fabrication of halide double perovskites. In the first place, more precursors are needed, and most of the halide salts (M^I^X and M^III^X_3_) exhibit poor solubility in solvents commonly used for lead-based perovskites, such as dimethylsulfoxide (DMSO) and *N,N*-dimethylformamide (DMF). In addition, the monovalent A-site cation (i.e., Cs^+^) has been found to be difficult to tune while satisfying the tolerance factor^24^ and achieving thermodynamic stability, limiting studies on the impact of the A-site on the optoelectronic properties in these materials. Similar challenges are associated with the synthesis of compositions where mixtures of monovalent metals M^I^ or halides can be used as an alternative route to achieve bandgap tunability of these materials. Whereas several compositions have been reported experimentally in the form of powders and single crystals,^13^ there are only a few examples of halide double perovskites in the form of thin films.^4,25,26^ Considering that thin films are most suitable for spectroscopic measurements such as transient absorption, photoluminescence, and time-resolved photoconductivity, it is not surprising that the most studied composition, Cs_2_AgBiBr_6_, coincides with the most soluble double-perovskite composition in DMSO. The solubility of the precursors is one of the main bottlenecks for rapidly obtaining a comprehensive understanding of this class of materials. Solid-state synthesis techniques (such as ball-milling or oven-based powder synthesis) may provide a route to circumvent this issue. Ball-milling involves vigorously shaking a vessel containing stainless steel balls that continuously bump with each other and the walls of the vessel, crushing and mixing the material within. During these collisions, depending on the type of mill and the operation frequency, a large amount of energy is transferred to the raw materials, intensifying the diffusion processes in solids and accelerating the chemical reactions. This allows these chemical reactions to be performed at low temperatures.^27^ The resulting double-perovskite powders can be dissolved and spin-coated or deposited on substrates using dry techniques such as physical vapor deposition (PVD)^28^ and pulsed laser deposition (PLD).^29^ These dry deposition techniques are useful to obtain near-stoichiometric transfer of multi-compound materials with any desired film thickness. Drawbacks of using solid-state synthesis techniques like ball-milling are the incomplete reaction of precursors and the poor control of the crystallite size, which is an important parameter for manipulating the optoelectronic properties of such materials. Thus, investigations on the synthesis products as a function of the milling conditions (i.e., frequency and time) and the effects of introducing chemical additives to control the kinetics of the crystallization should be conducted to make full use of this synthetic strategy. Another challenge in the solution-based fabrication of such halide double perovskites is that the low-dimensional, non-conductive, 3:2:9 phase (e.g., Cs_3_Bi_2_Br_9_) is thermodynamically favored,^30^ thus competing with the elpasolite phase (i.e., Cs_2_AgBiBr_6_). The 3:2:9 phase consists of face-sharing double-layered [Bi_2_Br_9_]^3–^ octahedra. A fingerprint for identifying the 3:2:9 phase is the presence in the X-ray diffraction pattern of the reflection^31^ at ∼8.7° (2θ), corresponding to the (001) plane. High-temperature synthesis of Cs_2_AgBiBr_6_ has been shown to lead to the formation of the 3:2:9 phase and elemental silver.^32^ This formation of Cs_3_Bi_2_Br_9_ is favored under bromide-poor conditions and during synthesis at high temperature (e.g., bottom-up synthesis). The presence of a reducing environment in combination with the low standard reduction potential of silver may facilitate the formation of elemental silver. In solution-based synthesis routes, effective strategies to control the formation of the desired composition rely on the control of the precursor stoichiometry; e.g., using an excess of bromine may suppress the formation of the 3:2:9 phase. Alternatively, performing the synthesis in an oxidative environment and/or carefully controlling the pH could also be used to hinder the formation of undesired phases. Despite the high-energy bandgap and low carrier mobility exhibited by 3:2:9 phases, such materials have been demonstrated to be promising candidates as photocatalysts for several reactions, such as ring-opening of epoxides,^33^ photodegradation of dyes,^34^ and photoreduction of carbon dioxide to carbon monoxide and methane at the gas–solid interface.^35^ Nevertheless, very few compositional variations of this 3:2:9 phase have been reported, most of them showing different metals at the bismuth position.^36^

To exploit a successful technological deployment of halide double perovskites, a comprehensive assessment of the fundamental properties as functions of the synthesis route and composition is vital. Of particular interest are understanding and controlling the role of defects, which have been shown to underpin the limitations in device operations as in any other semiconductor.^37,38^ Most of the reported halide double perovskites show weak and very broad photoluminescence spectra that are substantially red-shifted (up to 1 eV) with respect to the absorption onset. This is in sharp contrast with the lead-based perovskites that show strong, narrow photoluminescence at the band edge. Temperature-dependent photoluminescence and absorption have been used to get insight into the origin of the absorption features and broad photoluminescence spectra in halide double perovskites. Due to the bonding VBM and anti-bonding CBM of halide double perovskites, the absorption bandgap blue-shifts on lowering the temperature. In contrast, no change or minor red-shifts in photoluminescence have been observed for Cs_2_AgBiBr_6_, accompanied by a slight narrowing of the emission line width (∼20 meV).^39^ For some of the halide double perovskites (e.g., Cu_2_AgBiI_6_,^40^ Rb_4_Ag_2_BiBr_9_^41^), even more complex low-temperature spectra have been observed, showing multiple peaks also in the near-infrared region of the spectrum. The origin of the emission feature is still heavily under debate. The blue-shift of the absorption onset observed upon lowering the temperature, combined with the minor red-shift of the emission, suggests that likely these emission features do not share a common origin, and therefore, photoluminescence is not associated with band-to-band recombination of free charge carriers. Several mechanisms have been proposed, and the most common ones are schematically represented in Figure 2. One of the mechanisms proposes the dynamic formation of self-trapped charges or excitons (i.e., small polaron), promoted by the strong electron–phonon coupling, that subsequently diffuse to color centers^42^ (i.e., a vacancy occupied by an electron that gives rise to transitions that absorb and emit light in the visible spectrum), causing broad emission (Figure 2a). Related to defects, intervalley scattering has also been suggested as the origin of low-energy emission, where a rapid transition (∼10 ps) from an indirectly to a directly bound exciton leads to the recombination of indirectly bound excitons and electrons with trapped holes.^43^ The formation of such strongly bound excitons is promoted by the formation of stable shallow defects such as Ag^+^ vacancies (intrinsic defects) that leads to localization of holes in the valence band.^44^ Another origin of the emission in these materials could be the presence of stationary color centers (Figure 2b). Local inhomogeneities in the distribution of the M^I^ or M^III^ can also be responsible for the formation of local emissive states^45^ with sub-bandgap energies (Figure 2c). In fact, defect bands can be engineered by manipulating the distribution of the metals to produce local domains with different M^I^/M^III^ ratios.^46^ For example, whereas intermediate bandgaps are obtained on mixing, e.g., Bi^3+^ and In^3+^, the significant red-shift observed for the incorporation of only 1% iron (Figure 1b) would suggest the presence of a defect band or local domains with high concentrations of Fe^3+^/In^3+^ ratio rather than an intermediate bandgap. Such inhomogeneities could be interrogated at the nanoscale by optical, structural, and analytical techniques which include spatially resolved photoluminescence, time-of-flight secondary-ion mass spectrometry (TOF-SIMS),^47^ nano X-ray diffraction (nano-XRD), and electron back-scattering diffraction (EBSD).^48^ Spatially resolved photoluminescence could potentially probe local emissive domains within the perovskite film, but only if these are micrometer-sized. Another limitation of such photoluminescence-based techniques is related to the accumulation and recombination of charges in the low-energy emissive states, providing only a limited picture in the case of an inhomogeneous electronic landscape. TOF-SIMS can provide information about the uniformity of the elemental distributions through the depth of the film. However, one significant limitation is the complex relationship between the intensity of the signal and the concentration of the probed elements, which makes absolute quantification difficult. Nano-XRD can efficiently probe the existence of separated phases consisting of metal-enriched domains, provided that the resolution is sufficient to distinguish the diffraction signal of different metal-enriched domains. The local distribution of those inhomogeneities could be measured by high-resolution EBSD that allows for the identification of different crystal phases with high spatial resolution (∼10 nm at low current doses) and characterizations of the grains, their size, and their shape. EBSD could even discriminate between compounds with the same crystal structure but different elemental composition if combined with an energy-dispersive X-ray (EDX) detector. Interestingly, the spatially resolved approach of EBSD can be complementary to other local techniques to correlate the nanoscale structural and optical or electrical properties. In semiconductors, the presence of structural inhomogeneities and defects also has huge implications on the charge carrier mobility. In Cs_2_AgBiBr_6_ and similar materials, initially after photoexcitation, charges with decent mobilities (∼12 cm^2^/(V·s)) can be generated,^42^ but these lose mobility within tens of nanoseconds, leading to poor electron and hole transport.^25,49^ Defect engineering would make it possible to improve the majority carrier mobility of such materials, as already shown in vacancy-ordered perovskites^50^ such as Cs_2_SnI_6_. Here, it is hypothesized that *n*-type conductivity originates from iodine vacancies that serve as electron donors, leading to dark conductivities comparable to those in CsSnI_3_.^51^

**Figure 2:**
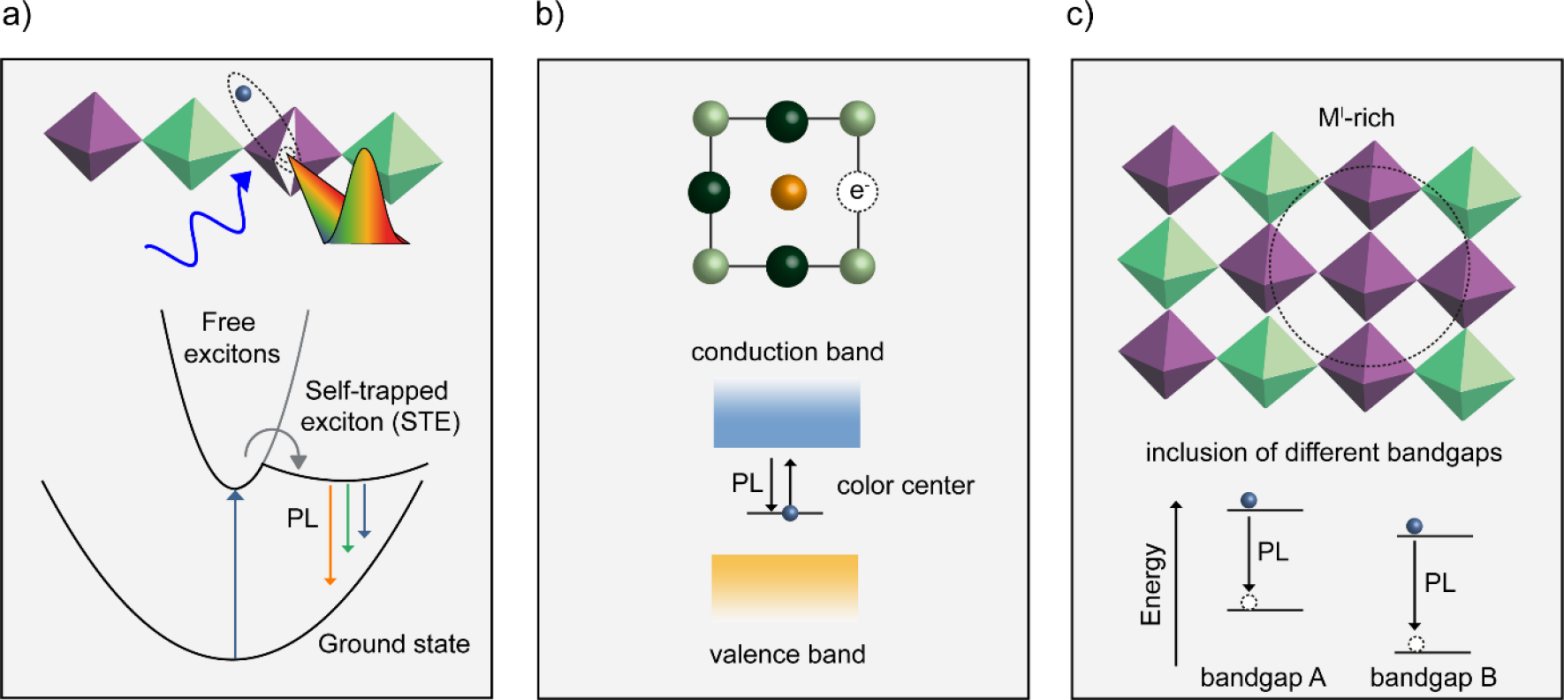
Schematic representation of the proposed mechanisms behind the origin of the photoluminescence in Cs_2_AgBiBr_6_ and similar materials. (a) Due to the high electron–phonon coupling, the photogenerated exciton could be trapped by the lattice in small polarons. These self-trapped excitons could then diffuse to a color center and emit. (b) The presence of a vacancy occupied by an electron could result in a transition that absorbs the light used for excitation and emits in the visible region of the spectrum. (c) Inhomogeneities in the metals distribution could result in the formation of local domains with different M^I^/M^III^ ratios (circled), and thus multiple emissive domains.

The suitable bandgap of Cs_2_AgBiBr_6_ for indoor PV,^52^ together with its improved stability and reduced toxicity compared to lead-based halide perovskites, makes it worth investigating whether trap densities in this material could be reduced to an acceptable value (i.e., less than 10^15^ cm^–3^). It would therefore be of interest to study this class of materials with ultrafast X-ray absorption spectroscopy to reveal the dominant defect state^53^ or to use highly sensitive X-ray techniques to probe local heterogeneities for different compositions and synthesis routes through extended X-ray absorption fine structure (EXAFS).^54^ Optimizing synthesis routes through varying the type of precursor, the solvent, the temperature, or the pressure, or by adding passivating molecules, could then ideally make it possible to prepare halide double perovskites with enhanced performance. However, it will be trickier for this class of materials than for lead-based halide perovskites, where most intrinsic defects do not result in intra-bandgap states. On the other hand, the presence of trap states may turn out to be beneficial for some of the envisioned applications of halide double perovskites. As an example, the non-mobile charges in Cs_2_AgBiBr_6_ have a spectacularly long lifetime, exceeding tens of microseconds.^55^ If these long-lived charges reside at the surface of the crystallites, these may, depending on their absolute energy, be used for photoredox chemistry. Although some promising first results have been obtained in this research area,^23^ the use of halide double perovskites for photoredox catalysis has been largely underexplored. The vast chemical space offered by halide double perovskites exhibits potential for a plethora of applications beyond photovoltaics, including lasers, photocatalysts, humidity and temperature sensors, memory devices, and X-ray detectors (Figure 3). The exciton binding energy on the order of a few hundred meV^56^ reported for Cs_2_AgBiBr_6_ and similar materials discourages applications where long-range transport is required, but local exciton separation can serve as a strategy to facilitate their use in photovoltaics and photocatalysis. Such local exciton separation could be achieved by using halide double perovskite nanocrystals decorated with metals or connected to metal–organic frameworks, or by making more complex structures or blends of donors/acceptors. In addition, further investigations are needed to assess the binding energy of compositions suitable for photovoltaics (e.g., Cs_2_AgFeCl_6_). On the other hand, high binding energies are promising for display and lighting applications (e.g., LEDs) where they, along with direct bandgaps, promote efficient radiative recombination. Another route toward highly emissive materials involves the introduction of a continuous bandgap gradient by local tuning of the composition, e.g., low-band-gap inclusions. This smart light management approach could enhance the photoluminescence quantum yield (PLQY), as already demonstrated for lead-based layered two-dimensional (2D) perovskites and segregated mixed-halide perovskites.^57^

**Figure 3:**
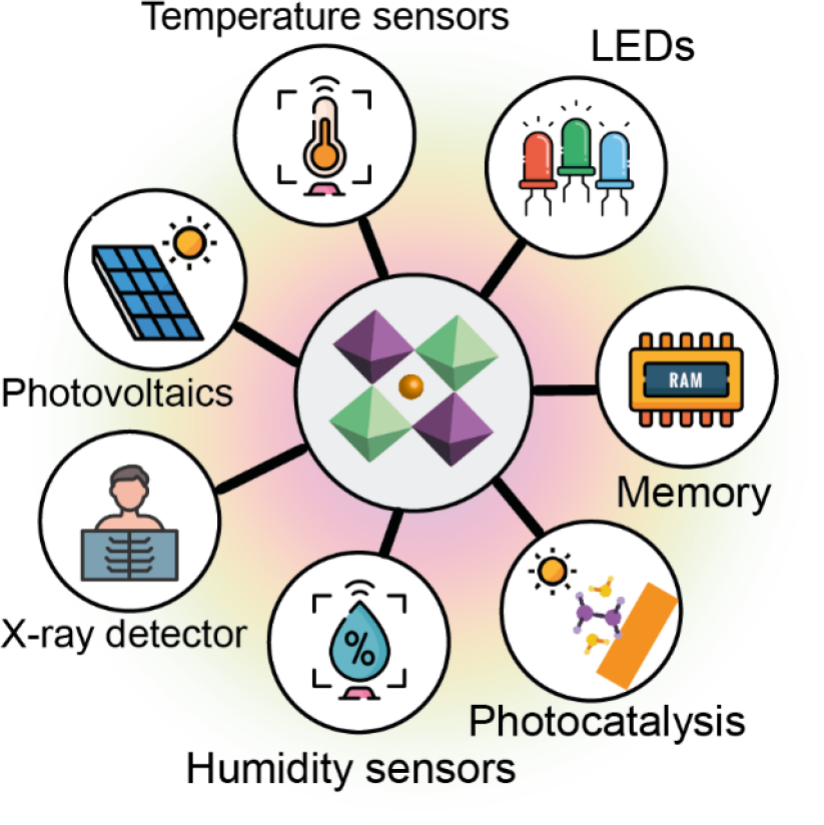
Schematic summary of the potential applications of halide double perovskites.

In general, the compositional variation of A_2_M^I^M^III^X_6_ reported so far is only the tip of the iceberg, and this class of materials offers enormous potential to design materials for targeted applications. One example is the design of (ferro)magnetic perovskites by incorporating metals such as iron,^58^ neodymium,^59^ nickel, or cobalt.^60^ Such materials could be relevant for next-generation high-speed and low-power-consumption information technology (i.e., spintronics) or for optomagnetic and magnetoelectric applications (e.g., sensors and memory devices), where the magnetization (or polarization) can be controlled by an external field.

Thermochromic applications could benefit from the strong electron–phonon coupling and spin–orbit coupling effects in halide double perovskites, paving the way toward the design of smart windows, temperature sensors, and visual thermometers.^61^ Yet, the heat transport properties of these materials are barely reported.^62^ Another exciting future route includes the incorporation of lanthanides in double halide perovskites.^63,64^ Lanthanides (Ln) have been well-known for their applications as highly sensitive temperature sensors, in lasing, and in non-linear optics such as up-conversion. Several examples of the incorporation of Ln^3+^ ions, such as Yb^3+^,^65^ Ho^3+^,^66^ and Eu^3+^,^67^ have already been reported. Colloidal quantum dots and nanostructures of the most promising halide double perovskites could be used to further tune the optoelectronic properties and the stability of such materials.^68^ Finally, although some 2D compositions have been recently reported,^69^ systematic and representative explorations on this class of materials are still largely underrepresented. We suspect that the incorporation of large organic moieties to induce the formation of two-dimensional (2D) halide double perovskites has huge potential, since specific functionalized spacers^70^ (e.g., photoactive, electroactive, chiral) that are responsive to various stimuli can serve as platforms for advanced functions in future smart nanotechnologies.^69^
